# Alterations in the gut microbiome and its metabolites are associated with the immune response to mucosal immunization with *Lactiplantibacillus plantarum*-displaying recombinant SARS-CoV-2 spike epitopes in mice

**DOI:** 10.3389/fcimb.2023.1242681

**Published:** 2023-08-29

**Authors:** In-Chan Hwang, Robie Vasquez, Ji Hoon Song, Lars Engstrand, Valerie Diane Valeriano, Dae-Kyung Kang

**Affiliations:** ^1^ Department of Animal Biotechnology, Dankook University, Cheonan, Republic of Korea; ^2^ Department of Microbiology, Tumor and Cell Biology, Centre for Translational Microbiome Research (CTMR), Karolinska Institutet, Stockholm, Sweden

**Keywords:** gut microbiome, mucosal vaccine, SARS-CoV-2, COVID-19, lactic acid bacteria, butyrate

## Abstract

Lactic acid bacteria (LAB) expressing foreign antigens have great potential as mucosal vaccines. Our previous study reported that recombinant *Lactiplantibacillus plantarum* SK156 displaying SARS-CoV-2 spike S1 epitopes elicited humoral and cell-mediated immune responses in mice. Here, we further examined the effect of the LAB-based mucosal vaccine on gut microbiome composition and function, and gut microbiota-derived metabolites. Forty-nine (49) female BALB/c mice were orally administered *L. plantarum* SK156-displaying SARS-CoV-2 spike S1 epitopes thrice (at 14-day intervals). Mucosal immunization considerably altered the gut microbiome of mice by enriching the abundance of beneficial gut bacteria, such as Muribaculaceae, *Mucispirillum*, Ruminococcaceae, *Alistipes, Roseburia*, and Clostridia vadinBB60. Moreover, the predicted function of the gut microbiome showed increased metabolic pathways for amino acids, energy, carbohydrates, cofactors, and vitamins. The fecal concentration of short-chain fatty acids, especially butyrate, was also altered by mucosal immunization. Notably, alterations in gut microbiome composition, function, and butyrate levels were positively associated with the immune response to the vaccine. Our results suggest that the gut microbiome and its metabolites may have influenced the immunogenicity of the LAB-based SARS-CoV-2 vaccine.

## 1 Introduction

Coronavirus disease 2019 (COVID-19) is a fatal respiratory disease caused by SARS-CoV-2 (Al-Jighefee et al., 2021). At the time of writing, the total global infection has reached 768 million since the start of the pandemic, with a fatality rate of approximately 0.9% (World Health Organization, 2023). Vaccination against the virus remains the most effective way to combat COVID-19, and several vaccines have since been developed, each designed to specifically target vulnerabilities in the viral life cycle of SARS-CoV-2 (Al-Jighefee et al., 2021). SARS-CoV-2 infection alters the host gut microbiome; conversely, the gut microbiome influences the disease severity and impacts host’s response to SARS-CoV-2 vaccines (Chen et al., 2021; Li et al., 2022; Pereira et al., 2022; Zhang et al., 2022a; SeyedAlinaghi et al., 2023). Moreover, recent evidence suggests that the gut microbiome is associated with post-acute COVID-19 syndrome (Liu et al., 2022). To further elucidate the role of the gut microbiome, several gut commensals that could potentially enhance vaccine immunogenicity have been identified (Ng et al., 2022; Tang et al., 2022). Interest in the role of gut microbiota-derived SCFAs, especially butyrate, in SARS-CoV-2 infection and vaccine immunogenicity is also increasing (Kuriakose et al., 2021; Li et al., 2021a).

Mucosal vaccination is a powerful strategy for priming the immune system against diseases (Neutra and Kozlowski, 2006; Lavelle and Ward, 2022). Compared to systemic vaccines, mucosal vaccines may provide more efficient and long-lasting protection against diseases, especially COVID-19 (Alturaiki, 2022; Miteva et al., 2022). However, mucosal vaccine candidates against COVID-19 are still under development (Miteva et al., 2022). Researchers have been exploring the use of lactic acid bacteria (LAB) as vectors for mucosal vaccines over the past decades (Wyszyńska et al., 2015). Owing to its non-pathogenic nature, Generally Regarded As Safe status, and potential probiotic benefits, LAB are great vector candidates for oral mucosal vaccines (Wyszyńska et al., 2015). For instance, candidate LAB-based mucosal vaccines have been designed and tested for human norovirus and papillomavirus-16 (Cortes-Perez et al., 2003; Craig et al., 2019). However, published reports on LAB-based mucosal vaccines against COVID-19 are minimal. Thus, inn our previous study, we designed and demonstrated the potential of *Lactiplantibacillus plantarum* SK156-displaying SARS-CoV-2 spike epitopes as mucosal vaccines against COVID-19 (Hwang et al., 2023). Immunization *L. plantarum* SK156-displaying SARS-CoV-2 with spike epitopes elicited humoral and cell-mediated immune responses in mice. However, we have yet to explore the effect and potential role of the gut microbiome in the immunogenicity of candidate mucosal vaccines. Thus, this study further examined the association of the gut microbiome and SCFA with the host’s immune response to mucosal immunization with LAB-displaying SARS-CoV-2 spike epitopes.

## 2 Materials and methods

### 2.1 Mice immunization strategy and sample collection

Forty-nine 7-week-old specific pathogen-free female BALB/c mice (Raonbio, Korea) were randomly divided into six groups (Control, n=5; SK156, n=5; S1-1, n=10; S1-2, n=10; S1-3, n=9; S1-4, n=10) after one week of acclimatization to laboratory conditions. The laboratory conditions were maintained at 45–50% relative humidity and 22–25°C, with a 12-h light-dark period. Mice were provided *ad libitum* access to a standard pellet diet containing 4 kcal/g of protein, 9 kcal/g of fat, and 4 kcal/g of available carbohydrate (2018S Teklad Global 18% Protein Rodent Diet; Envigo, USA) and filtered tap water. The construction of a recombinant *L. plantarum* SK156 surface displaying SARS-CoV-2 spike S1 epitopes is detailed in our previous study (Hwang et al., 2023) (**Supplementary Figure S1**). Oral immunizations were performed on days 0 (priming), 14 (1^st^ booster), and 28 (2^nd^ booster) by feeding 100 µL solution containing phosphate-buffered saline (PBS) only (pH 7.4; Control), 1.2 × 10^9^ CFU wild-type *L. plantarum* SK156 in PBS (SK156 group), or 1.2 × 10^9^ CFU recombinant *L. plantarum* SK156 surface displaying SARS-CoV-2 spike S1 epitopes (S1-1, S1-2, S1-3, or S1-4) (**Supplementary Figure S2**). Aside from its ability to induce strong systemic and mucosal immune responses and its ease of use (Wang et al., 2015; Kar et al., 2022), oral route was chosen to examine the effects of the LAB-based vaccine on the gut microbiome after immunization. Blood and fecal samples were collected on days 0, 21, and 35 for immunological analysis. SCFA concentrations (acetate, butyrate, and propionate) were also determined from fecal samples. All mice were euthanized under CO_2_ gas on day 35, and ceca were carefully collected for metagenomic sequencing. All samples were immediately stored at -70°C after collection until further analysis. All animal experiments were approved by the Dankook University Ethics Committee (DKU-20-053).

### 2.2 Antigen-specific IgG/IgA and cytokine detection using enzyme-linked immunosorbent assay

Interleukin (IL)-4, IL-10, and interferon (IFN)-γ in the serum of immunized mice were detected using commercial enzyme-linked immunosorbent assay (ELISA) kits (R&D Systems, Minneapolis, MN, USA). Standard curves were generated for each cytokine. Values were measured at an optical density of 450 nm using an ELISA plate reader (SpectraMax M2e, Molecular Devices, San Jose, CA, USA). Antigen-specific immunoglobulin (Ig)G from serum or IgA from fecal samples was measured using ELISA based on our previous study (Hwang et al., 2023). Briefly, each well of a 96-well plate was pre-coated with *E. coli*-expressed recombinant SARS-CoV-2 spike S1 antigens (1 µg per well) overnight at 4°C. The antigen-coated wells were blocked with bovine serum albumin for 1 h at 37°C. After blocking, immunized mouse serum (1:100 dilution) or fecal extract (1:10 dilution) was added to the wells and incubated at 37°C for 1 h. HRP-conjugated goat anti-mouse IgG or IgA antibody (1:1000; Invitrogen, Waltham, MA, USA) was added to the wells and incubated for 1 h at 37°C. The antigen-specific antibody was detected by adding 3, 3’, 5, 5’- tetramethylbenzidine substrate (Sigma-Aldrich, St. Louis, MO, USA). The reaction was stopped by adding 0.5 N H_2_SO_4_. Optical density (450 nm) was measured using an ELISA plate reader (SpectraMax M2e, Molecular Devices, San Jose, CA, USA).

### 2.3 Next-generation sequencing and microbial community analysis

Based on the manufacturer’s instructions, genomic DNA from cecal samples was extracted using the QiaAmp PowerFecal Pro DNA Kit (Qiagen, Hilden, Germany). Blanks were used to check for any contamination during DNA extraction, which was performed with agarose gel electrophoresis. Library construction and sequencing of the V3–V4 hypervariable region of the 16S rRNA with Illumina MiSeq (2 × 250 bp paired-end; Illumina, CA, USA) was performed according to the manufacturer’s instructions (CJ BioScience, Inc., Korea). The primers used to amplify the V3-V4 region are summarized in **Supplementary Table S1**. The quality of the 16S rRNA library was checked through agarose gel electrophoresis. Raw sequence data generated by the 16S rRNA gene were processed using the Quantitative Insights Into Microbial Ecology pipeline (QIIME2, version 2022.8) (Bolyen et al., 2019). Primers and adapters were removed from the raw sequences using the ‘cutadapt’ plugin (Martin, 2011). Sequence quality control and feature table construction were performed using DADA2 (Callahan et al., 2016). Phylogenetic diversity and alpha rarefaction analyses were performed with ‘q2-phylogeny’ and ‘q2-diversity’, respectively. The feature classifiers were trained by ‘q2-feature-classifier’ within QIIME2, using the SILVA 138_99 database (Quast et al., 2013). Mitochondria and chloroplast 16S rRNA sequences were filtered-out. Principal coordinate analysis (PCoA)-based Bray–Curtis distance matrix and weighted uniFrac were performed in QIIME2. Alpha diversity indices, PCoA plots, and relative abundance bar graphs were visualized in the R program v.4.0.2 (Core R Team, 2019).

Differential taxonomic markers for each group were determined using ‘run_lefse’ package in the R program based on Linear discriminant analysis effect size (LEfSe) (Segata et al., 2011). Functional prediction using the Kyoto Encyclopedia of Genes and Genomes (KEGG) database was performed using Phylogenetic Investigation of Communities by Reconstruction of Unobserved States (PICRUSt2) (Douglas et al., 2020). Correlation analyses were performed by calculating Pearson’s correlation coefficient with the ‘Hmisc’ package, then visualized using the ‘pheatmap’ package in the R program (Core R Team, 2019).

### 2.4 Determination of fecal SCFAs using high-performance liquid chromatography

Fecal samples were prepared to determine SCFAs (acetate, propionate, and butyrate). Briefly, 0.5 g fecal samples were suspended in 1 mL of sterile demineralized water and vortexed for 3 min. The supernatant was collected after centrifugation at 10,000 × *g* for 10 min at 4°C, then filtered through 0.22-μm PTFE syringe filters. Samples were injected into an Agilent Infinity 1260 HPLC System (Agilent, Santa Clara, CA, USA) with an Aminex HPX-87H column with dimensions of 300 × 7.8 mm (Bio-Rad, Hercules, CA, USA). Signals were detected using a UV detector set at λ = 210 nm. Samples (10 μL) were injected using an autosampler. The mobile phase used was 0.005 M H_2_SO_4_, and the column temperature was 65°C. The flow rate was maintained at 0.6 mL/min for a total run time of 35 min per sample.

### 2.5 Statistical analyses

Statistical analyses of microbial compositions and SCFA concentrations were performed using the R program version 4.0.2 (Core R Team, 2019), and the normality of the data distribution was analyzed using the Shapiro–Wilk test. One-way ANOVA with Tukey’s *post hoc* test was used to analyze significant differences among the treatment groups. False discovery rate (FDR) correction was performed as necessary. Kruskal–Wallis tests for alpha and beta diversity were performed within the QIIME2 pipeline. Permutational multivariate analysis of variance (PERMANOVA) was used to determine significant differences in the PCoA plot. All statistical analyses were considered significant at (*p* < 0.05).

## 3 Results

### 3.1 Changes in gut microbiome after mucosal vaccination are associated with antibody and cytokine response

Sequencing of DNA extracted from the mouse ceca generated 7,253,331 paired-end reads for 49 samples, with an average read of 148,026 ± 25,511 reads per sample (**Supplementary Table S2**). After chimeras were filtered and low-quality sequences were removed, 3,676,495 valid reads were obtained, with an average read length of 411.5 bp. Rarefaction curves using alpha diversity index (Chao1) showed that the sequences obtained were sufficient for further data analyses (**Supplementary Figure S3**). All sequences generated and analyzed in this study were deposited and publicly available in the National Center for Biotechnology Information (NCBI) database, with accession number PRJNA954075.

**Figure 1:**
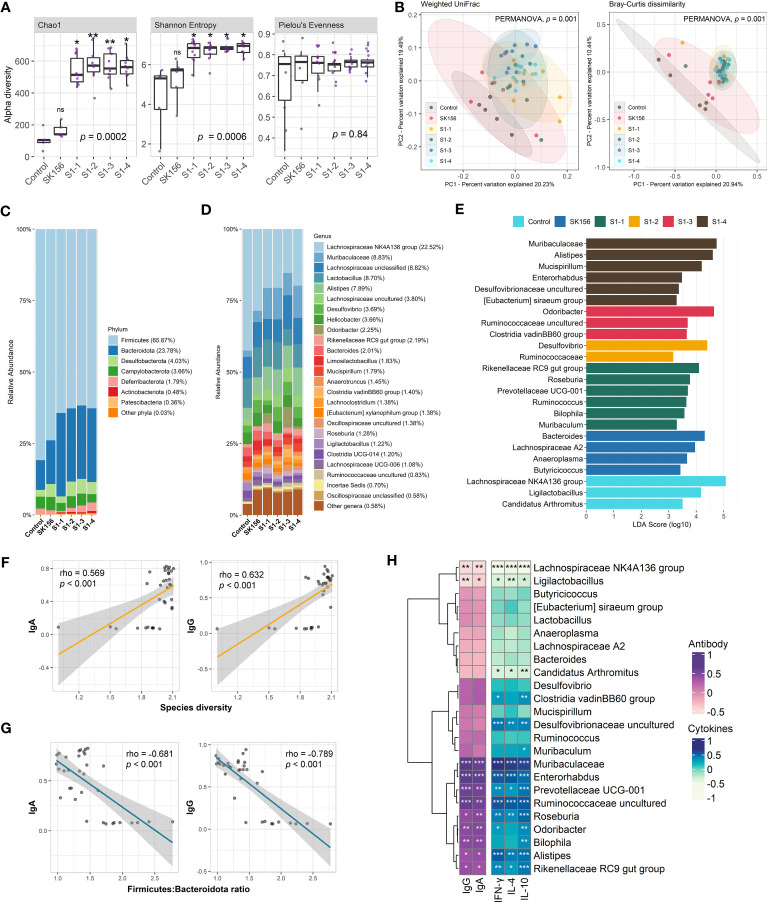
Changes in the gut microbiome after immunization with LAB-based mucosal vaccine. **(A)** Changes in alpha diversity: Chao1, Shannon, and Pielou's evenness. **(B)** Principal coordinate analysis (PCoA) showing Bray–Curtis distance matrix and weight uniFrac. Relative abundance of the gut microbiome at phylum **(C)** and genus **(D)** levels. **(E)** Taxonomic marker identification using Linear Differential Analysis Effect Size (LEfSe). Association between the antibody (IgA and IgG) response with species diversity **(F)** and Firmicutes:Bacteroidota ratio **(G)**. Association between antibody response and cytokine levels (IL-4, IL-10, IFN-γ) with taxonomic markers **(H)**. Significant differences (alpha diversity) were calculated using Kruskal–Wallis with *post hoc* Dunn's multiple comparisons tests. Correlation (rho) and p values were calculated using Pearson's correlation with false discovery rate (FDR). *P* values are denoted by *, **, and ***, at *p* < 0.05, *p* < 0.01, and *p* < 0.001, respectively. ns is not significant.

Using QIIME2, we measured alpha and beta diversity changes in control and immunized mice. We observed changes in alpha diversity indices, especially for species richness and diversity (**Figure 1A**). Species richness (Chao1) and diversity (Shannon Entropy) significantly increased in immunized mice compared to the Control or SK156 groups (*p* < 0.001). However, no significant differences among the immunized groups (S1-1 to S1-4) for Chao1 and Shannon were observed (*p* > 0.05; **Supplementary Table S3**). Furthermore, we did not observe a significant difference in species evenness among groups (*p* > 0.05), as measured by Pielou’s evenness. PCoA plots based on Bray–Curtis dissimilarity and weighted uniFrac (**Figure 1B**) showed distinct clustering of groups S1-1 to S1-4 from the Control and SK156 groups, indicating that the mucosal vaccine significantly modulated the gut microbiome composition (PERMANOVA, *p* = 0.001; **Supplementary Tables S4, S5**).

The composition of the murine gut microbiota was investigated at the phylum and genus levels. QIIME2 identified 118 bacterial genera in all samples. At the phylum level (**Figure 1C**, **Supplementary Table S6**), Firmicutes (65.87%) and Bacteroidota (formerly Bacteroidetes; 23.78%) dominated the gut across the groups. Firmicutes were significantly more abundant in the Control group than in vaccinated mice (*p* = 0.004). In contrast, Bacteroidota were significantly more abundant in the immunized group than in the Control group (*p* = 0.005). Consequently, we observed that the Firmicutes : Bacteroidota ratio was significantly decreased in immunized mice compared with the Control or SK156 groups (*p* = 0.006; **Supplementary Figure S4**). At the genus level, the Lachnospiraceae NK4A136 group, Muribaculaceae, unclassified Lachnospiraceae, *Lactobacillus*, and *Alistipes* were the major genera across the groups (**Figure 1D**, **Supplementary Table S5**). *Alistipes* and *Roseburia* were significantly enriched in the immunized groups (*p* = 0.01 and *p* = 0.009, respectively). In contrast, the abundance of *Bacteroides* was reduced in immunized mice (*p* = 0.04).

To further examine the influence of mucosal immunization on the gut microbiota composition, we identified the taxonomic markers of each group using LEfSe (**Figure 1E**). LEfSe revealed that Rikenellaceae RC9 gut group, *Roseburia*, Prevotellaceae UCG-001, *Ruminococcus*, *Bilophila*, and *Muribaculum* were differentially enriched in S1-1. While immunization with S1-2 enriched *Desulfovibrio* and family Ruminococcaceae. S1-3 had enriched populations of *Odoribacter*, uncultured Ruminococcaceae genus, and Clostridia vadinBB60 group; while Muribaculaceae, *Alistipes*, *Mucispirillum, Enterorhabdus*, uncultured Desulfovibrionaceae genus, and *Eubacterium siraeum* group were differentially enriched by S1-4 immunization. By contrast, *Bacteroides*, Lachnospiraceae A2, *Anaeroplasma*, and *Butyricicoccus* were significantly more abundant in SK156. Finally, Lachnospiraceae NK4A136 group, *Ligilactobacillus*, and *Candidatus* Arthromitus were abundant in the control group.

Next, we examined the association of changes in the gut microbiome composition with the immune response of the mice to mucosal immunization, particularly with IgA- and IgG-specific neutralizing antibodies and cytokines IL-4, IL-10, and IFN-γ. We observed that gut bacterial diversity across the groups (Shannon index) was significantly correlated with higher IgA and IgG responses (*p* < 0.001; **Figure 1F**). In contrast, the Firmicutes : Bacteroidota ratio, especially in immunized groups (S1-1, S1-2, S1-3, and S1-4) showed a strong negative association with IgA and IgG responses (*p* < 0.001; **Figure 1G**).

In addition, the association of enriched microbiota with IgA, IgG, IL-4, IL-10, and IFN-γ was also examined (**Figure 1H**). Pearson’s correlation analysis revealed that the population of immunization-enriched microorganisms such as Muribaculaceae (enriched in S1-4), *Enterorhabdus* (S1-4), Prevotellaceae UCG-001 (S1-1), uncultured Ruminococcaceae (S1-3), *Roseburia* (S1-1)*, Odoribacter* (S1-3)*, Bilophila* (S1-1)*, Alistipes* (S1-4), and Rikenellaceae RC9 gut group (S1-1) had a strong association with higher IgA, IgG, and IL-4, IL-10, and IFN-γ responses. Moreover, the Clostridia vadinBB60 group and uncultured Desulfovibrionaceae (enriched by S1-3 and S1-4, respectively) were positively correlated with elevated IL-4, IL-10, and IFN-γ titers. Conversely, antibody and cytokine responses were negatively associated with the population of Lachnospiraceae NK4A136, *Ligilactobacillus*, and *Candidatus* Arthromitus (enriched in Control group). Our results demonstrate that the composition of the murine gut microbiome is associated with the immune response to the LAB-based SARS-CoV-2 mucosal vaccine.

### 3.2 Gut microbiota-derived metabolites (SCFAs) are associated with the immune response

**Figure 2:**
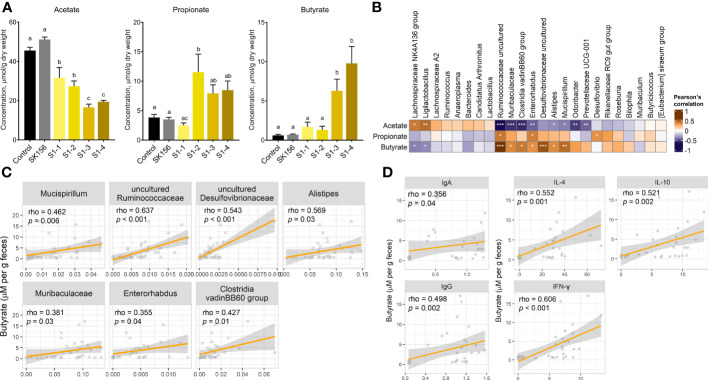
Changes in fecal short chain fatty acid (SCFA) concentrations after immunization with LAB-based mucosal vaccine. **(A)** Fecal concentrations of acetate, propionate, and butyrate. Lowercase letters indicate significant differences between groups. **(B)** Association between SCFAs and taxonomic markers. **(C)** Association between butyrate and gut microbiota. **(D)** Association of butyrate levels with antibody (IgA and IgG) and cytokine levels (IL-4, IL-10, IFN-γ). Significant differences were calculated using Kruskal–Wallis with *post hoc* Dunn's multiple comparisons tests. Correlation (rho) and *p* values were calculated using Pearson's correlation with false discovery rate (FDR). P values are denoted by *, **, and ***, at *p* < 0.05, *p* < 0.01, and *p* < 0.001, respectively.

To investigate the effect of mucosal immunization on gut microbiota-derived metabolites, we measured the fecal concentration of SCFAs after LAB-based SARS-CoV-2 mucosal vaccination (**Figure 2A**). The propionate concentration increased in groups S1-2, S1-3, and S1-4, but most drastically in S1-2 (*p =* 0.007 and *p* = 0.005 compared with Control and SK156, respectively). Whereas butyrate was drastically elevated in S1-3 and S1-4 compared to the Control (*p* = 0.018 and *p* = 0.002) or SK156 (*p* = 0.07 and *p* = 0.009) groups. In comparison, acetate concentration decreased in immunized groups, especially in S1-3 (*p* < 0.001) and S1-4 (*p* < 0.001), compared to either the Control or SK156 group. Correlative analysis between taxonomic markers and fecal SCFA concentrations was also performed. We observed a robust and positive association between butyrate and the abundance of uncultured Desulfovibrionaceae, uncultured Ruminococcaceae, *Alistipes*, *Mucispirillum*, Clostridia vadinBB60 group, Muribaculaceae, and *Enterorhabdus* (**Figures 2B, C**). Whereas the fecal concentration of propionate was positively correlated with increasing uncultured Ruminococcaceae, *Enterorhabdus*, and *Desulfovibrio* populations (**Supplementary Figure S5**). Notably, most microorganisms associated with propionate and butyrate were negatively associated with acetate levels. Moreover, the butyrate level was positively correlated with the immune response of the mice to the candidate mucosal vaccine (**Figure 2D**). Butyrate strongly correlated with higher IgA and IgG (*p* = 0.04 and *p* = 0.002, respectively) and IL-4, IL-10, and IFN-γ (*p* = 0.001, *p* = 0.002, and *p* < 0.001, respectively). Our results suggest that butyrate production by the gut microbiota may play an essential role in the immune response of mice to the LAB-based SARS-CoV-2 mucosal vaccine.

### 3.3 The gut microbiome function is associated with immune response

**Figure 3:**
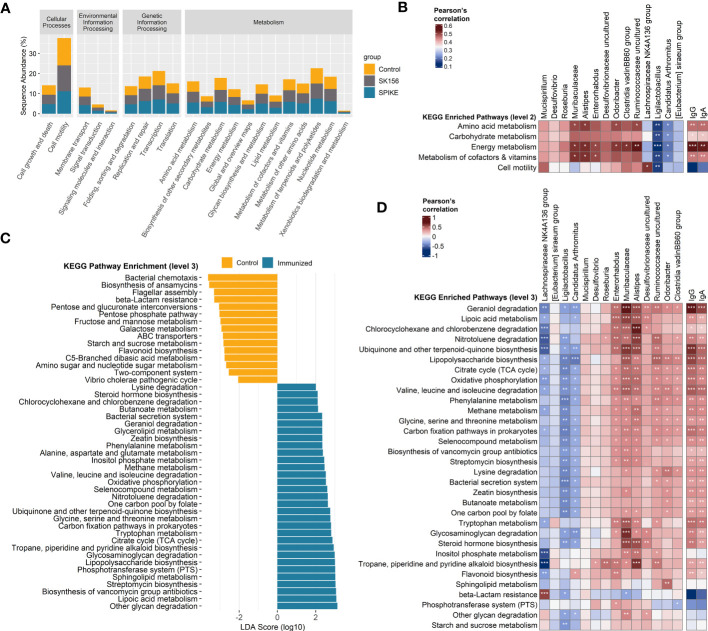
Inference of the gut microbiome function using PICRUSt2 and Kyoto Encyclopedia of Genes and Genomes (KEGG). **(A)** Abundance (log10) of KEGG pathways at level 2. **(B)** Association of altered KEGG level 2 pathways with the taxonomic markers and antibody-specific IgA and IgG response. **(C)** Functional marker identification using Linear Differential Analysis Effect Size (LEfSe). Association among the identified functional markers, taxonomic markers, and antibody (IgA and IgG) response. **(D)** Association of altered KEGG level 3 pathways with the taxonomic markers and antibody (IgA and IgG) response. Correlation (rho) and *p* values were calculated using Pearson’s correlation with false discovery rate (FDR). *P* values are denoted by *, **, and ***, at *p* < 0.05, *p* < 0.01, and *p* < 0.001, respectively. ns, not significant.

We examined the changes in the inferred function of the murine gut microbiome using the PICRUSt2 and KEGG database. We collectively compared the immunized groups (S1-1, S1-2, S1-3, and S1-4) with the Control and SK156 groups. Similar to the microbiome composition, the function of the gut microbiome was also significantly altered by immunization (*p* = 0.001; **Supplementary Figure S6**). At KEGG level 2, most pathways were enriched in immunized groups compared to the Control or SK156 groups (**Figure 3A**), most notably pathways for amino acid metabolism, carbohydrate metabolism, energy metabolism, and metabolism for cofactors and vitamins. We observed that these enriched pathways correlated positively with the abundance of Muribaculaceae, *Alistipes, Enterorhabdus, Odoribacter*, Clostridia vadinBB60 group, and uncultured Ruminococcaceae, and a higher antibody response (**Figure 3B**).

We further examined the predicted function of the gut microbiome at KEGG level 3. LEfSe analysis revealed that LAB-based mucosal immunization enriched several pathways, including other glycan degradation, lipoic acid metabolism, sphingolipid metabolism, lipopolysaccharide (LPS) biosynthesis, glycosaminoglycan degradation, tryptophan metabolism, ubiquinone and other terpenoid-quinone biosynthesis, valine, leucine, and isoleucine degradation, citrate cycle (TCA cycle), selenocompound metabolism, alanine, aspartate, and glutamate metabolism, geraniol degradation, and lysine degradation, and butanoate metabolism (**Figure 3C**). By contrast, bacterial chemotaxis, biosynthesis of ansamycins, flagellar assembly, and beta-lactam resistance, among others, were enriched in non-immunized mice. No functional markers were identified in the SK156 group. Furthermore, we observed that geraniol degradation, lipoic acid metabolism, LPS biosynthesis, valine, leucine, and isoleucine degradation, lysine degradation, butanoate metabolism, tryptophan metabolism, and glycosaminoglycan degradation were associated with the abundance of Muribaculaceae, *Alistipes, Enterorhabdus, Odoribacter*, Clostridia vadinBB60 group, and uncultured Desulfovibrionaceae and Ruminococcaceae (**Figure 3D**). These pathways were also strongly associated with specific IgA and IgG responses against the LAB-based mucosal vaccine (**Figure 3D**, **Supplementary Figure S5**). Our data shows that changes in the function of the gut microbiota are associated with the immune response to the LAB-based mucosal vaccine.

## 4 Discussion

We observed a substantial alteration in alpha and beta diversity of the gut microbiome composition after mucosal immunization with *L. plantarum* displaying SARS-CoV-2 spike S1 epitopes. This result is congruent with the knowledge that vaccine administration can alter the host gut microbiome (Guo et al., 2020; Tang et al., 2022). Furthermore, in this study, mucosal immunization increased the gut microbial diversity, and positively correlated with specific antibody responses in mice. The diverse composition of the gut microbiota signals gut stability and health which is essential in vaccine immunogenicity (Lynn et al., 2018; Lynn et al., 2022). However, several studies reported unaltered or diminished microbial diversity after vaccination, which is contradictory to our results (Eloe-Fadrosh et al., 2013; Hagan et al., 2019; Tang et al., 2022). Herein, elevated microbial diversity may potentially be due to the increase in the population of rare taxa which are not typically abundant in the absence of an immune stimuli, as observed elsewhere (Seekatz et al., 2013; Shen et al., 2023). IFN-elevated secretory IgA and systemic IgG could also increase the microbial diversity of the gut (Zeng et al., 2016; Yang and Palm, 2020; Ishizaka et al., 2023), consistent with our results.

In this context, the increase in gut microbiome diversity after mucosal immunization could be driven by changes in the Firmicutes and Bacteroidota phyla abundances. The abundances of these phyla are crucial microbiological markers of COVID-19 severity and immunization response since these bacterial groups influence angiotensin-converting enzyme-2 (ACE2) expression (Zuo et al., 2020; Nejadghaderi et al., 2021). A higher Firmicutes to Bacteroidota ratio is associated with a lower spike-specific CD4+ T cell response, indicating a lower response to COVID-19 vaccination, agreeing with our observation. In addition, an overpopulation of Firmicutes has been observed in patients with severe COVID-19 symptoms. Enrichment of Firmicutes is also prevalent after administering COVID-19 vaccines, attributing to severe inflammation after vaccination (Jiao et al., 2022; Ng et al., 2022). On the other hand, an interesting explanation for the enrichment of phylum Bacteroidota comes from Groves et al. (2018), who observed increased Bacteroidota abundance after vaccination with live attenuated influenza virus in mice. The authors hypothesized that mucus production by the host (as a response to the vaccine) provided an advantage to members of this phylum, especially Muribaculaceae, as this group of bacteria can potentially degrade mucus as an energy source. In our study, IFN-γ induction was only seen in S1-immunized groups which are known to directly stimulate mucus release (Farin et al., 2014). We also observed abundant mucus-degrading bacteria in immunized mice, supporting this hypothesis. Aside from Muribaculaceae, mucus-degrading *Mucispirillum* and *Enterorhabdus* growth was also favored in immunized mice, all of which positively correlated with pathways related to mucus degradation (glycosaminoglycan and other glycan degradation) and higher immune antibody titers. Glycans are non-digestible polysaccharides metabolized by gut microbes to produce SCFAs as byproducts (Preidis et al., 2015; Loy et al., 2017). *Mucispirillum* is a mucus-associated commensal responsible for activating T-cell-dependent IgA production (Bunker et al., 2015; Loy et al., 2017) and protecting the host from *Salmonella* infection (Herp et al., 2019). *Enterorhabdus* is an SCFA producer via carbohydrate metabolism (Bagheri et al., 2022; Ma et al., 2022) and is depleted in patients with COVID-19, indicating its potential beneficial role in gut homeostasis (Al Bataineh et al., 2021; SeyedAlinaghi et al., 2023).

*Alistipes, Roseburia, Desulfovibrio*, and unclassified Desulfovibrionaceae were enriched in immunized mice. Owing to their potential beneficial roles, the genera *Alistipes* and *Roseburia* have been consistently identified in COVID-19-related studies. Not only are these commensals negatively associated with COVID-19 severity, but they are also associated with high responses to COVID-19 vaccines in various cohort studies (Zuo et al., 2020; Li et al., 2021b; Moreira-Rosário et al., 2021; Li et al., 2022; Ng et al., 2022; Alexander et al., 2023). Zhou et al. (2022) noted that the role of *Alistipes* in tryptophan metabolism may contribute to its beneficial role during SARS-CoV-2 immunization. Metabolites derived from tryptophan catabolism are crucial for attenuating the severity of COVID-19 (Belladonna and Orabona, 2020; Yokoyama et al., 2022). This study noted an association between *Alistipes* and tryptophan metabolism and a high vaccine response to mucosal immunization, consistent with other reports. On the other hand, *Roseburia* is associated with a better response to COVID-19 vaccination owing to its ability to prime mucosal immunity via fimbriae and butyrate production (Liu et al., 2022; Zhang et al., 2022a; Zhang et al., 2022b). *Desulfovibrio* species are LPS producers typically associated with inflammation, such as macrophage activation (Edlund et al., 1985; Duque and Descoteaux, 2014; Zhang et al., 2022b). However, LPS from commensal bacteria can act as an adjuvant and enhance vaccine immunogenicity by potent toll-like receptors (TLR) activation, such as TLR-4 (Alu et al., 2022; Brueggeman et al., 2022; Lynn et al., 2022). The upregulation of LPS biosynthesis after mucosal immunization may increase the immune response to the candidate mucosal vaccine, as demonstrated by its association with a higher antibody response.

Microbiota-derived metabolites, such as SCFAs, play a key role in regulating host’s antiviral response (Wirusanti et al., 2022). SCFAs, including acetate, propionate, and butyrate, are metabolites produced by anaerobic microorganisms primarily from carbohydrate metabolism and, to some extent, amino acids (Morrison and Preston, 2016; Ríos-Covián et al., 2016). Upon immunization, we observed an increase in butyrate levels, which coincided with higher antibody responses and IL-4, IL-10, and IFN-γ production. The role of butyrate in influencing immune response is well established. Butyrate can act as an anti-inflammatory agent via peroxisome proliferator-activated receptor (PPAR)-γ activation, NF-κB pathway suppression, and G-protein coupled receptors activation (Singh et al., 2014; Zhang et al., 2016; Kim et al., 2021; Föh et al., 2022; Włodarczyk et al., 2022). On the other hand, butyrate can modulate proinflammatory response via GPR41/43 activation which enhances IFN-γ production of CD4+ T cells, thereby increasing antiviral responses (Kespohl et al., 2017; Brown et al., 2022). Aside from IFN-γ, butyrate also upregulates the expression of other antiviral proteins against SARS-CoV-2 via TLR signaling pathways (Li et al., 2021a). Conversely, the expression of critical molecules for SARS-CoV-2 infection, ACE2, and transmembrane protease serine-2 is downregulated by butyrate (Li et al., 2021a). Due to the bidirectional relationship between the gut microbiome and host’s antiviral response, a proinflammatory milieu can, in turn, elevate the abundance of butyrate-producing commensals (Serena et al., 2017). Consequently, no notable impact on the microbiome, butyrate level, or IFN-γ level was observed on the wild type *L. plantarum*, suggesting that *L. plantarum* alone did not activate proinflammatory responses that may result in drastic changes in the microbiome composition and butyrate production. This leads us to believe that in this study, IFN-γ induction is activated only by the S1 protein epitopes displayed on *L. plantarum* sensed by pattern recognition receptors (PRRs) on sentinel cells such as macrophages and dendritic cells. The gut microbiota then responds to the inflammatory signaling cascade by producing SCFAs to modulate homeostatic IFN expression (Wirusanti et al., 2022). In this context, butyrate produced by the gut microbiome plays an important role in host/microbiome crosstalk and immunomodulation, especially during inflammatory conditions that may be consequential during infection. In patients with COVID-19, butyrate levels were reportedly lower than in uninfected patients, attributed to the depletion of SCFA-producing commensals (Zhang et al., 2022b). On the other hand, Tang et al. (2022) reported higher butyrate levels in high responders to BBIBP-CorV vaccination, indicating the beneficial role of butyrate- and butyrate-producing commensals in enhancing the immunomodulatory response to SARS-CoV-2 vaccines. In current literature, certain *in vitro* models have demonstrated the pro-viral effects of exogenous butyrate in viral replication due to their known role as a histone deacetylase inhibitor (HDAC) and effects on the type I IFN response (Chemudupati et al., 2020). However, exogenous administration of butyrate has also been shown to enhance immune responses to various vaccines (Sim et al., 2018; Cait et al., 2021; Wu et al., 2023). These contradicting effects may be due to the different types of SCFA administered, viruses and immortalized cell lines tested (Wirusanti et al., 2022), but certainly highlights the importance of microbiome experimental models and the understanding of the gut microbiota and their derived metabolites in immunoregulation. The depletion of butyrate-producing commensals negatively affects the gut-lung axis (Chen et al., 2021; Li et al., 2021b); thus, potentially raising the possibility of hyperinflammatory acute respiratory distress syndrome (ARDS) during COVID-19 infection (Wang et al., 2022), and the potential of an LAB-based mucosal vaccine which can tune immune responses through the gut microbiome. Members of the phylum Bacteroidota (Muribaculaceae, *Mucispirillum*, and *Alistipes*), family Ruminococcaceae (*Ruminococcus* and *Roseburia*), and several clostridial groups are crucial butyrate-producing bacteria in the gut. Reduction of these gut commensals, especially Bacteroidota, Ruminococcaceae, *Roseburia*, and *Alistipes*, is associated with decreased butyrate levels, COVID-19 severity, attenuated vaccine response, and long-term COVID-19 infection (Moreira-Rosário et al., 2021; Alharbi et al., 2022; Alexander et al., 2023). Thus, enrichment of butyrate-producing bacteria and butyrate may enhance the host’s antiviral response resulting in a positive response to mucosal immunization against SARS-CoV-2.

The function of the microbiome in the gut-lung axis during SARS-CoV-2 infection is perturbed (Haiminen et al., 2021; Li et al., 2021b; Xu et al., 2021; Pereira et al., 2022; Rafiqul Islam et al., 2022). However, immunization against COVID-19 also results in altered gut microbiome functions (Ng et al., 2022; Tang et al., 2022). Here, we observed an alteration of gut microbiome function upon administration of *L. plantarum* displaying the SARS-CoV-2 spike epitopes. Similar to previous data, these changes are also associated with the immune response to the vaccine. We detected considerable increases in amino acid metabolism, energy metabolism, metabolism of cofactors and vitamins, and carbohydrate metabolism. This finding is concordant with the data observed by Zhang et al. (2022c) after oral immunization with yeast-displaying SARS-CoV-2 spike protein RBD, indicating the importance of these microbial functions in immunization. Furthermore, strong correlations were observed between level 3 pathways and higher antibody responses to mucosal immunization. For example, geraniol degradation, lipoic acid metabolism, ubiquinone and other terpenoid-quinone biosynthesis, and valine, leucine, and isoleucine degradation are associated with the immune response to immunization. Upregulation of pathways for geraniol degradation has been observed in patients with ulcerative colitis (Zhu et al., 2022), whereas ubiquinone and other terpenoid-quinone biosynthesis pathways have been reported in chicks infected with *Salmonella* Typhimurium (Shao et al., 2022). These pathways may signal positive responses to immune stimuli and counterbalance inflammation (Medicherla et al., 2015; Duan et al., 2021; Shao et al., 2022). The metabolism of branched-chain amino acids (BCAAs), such as valine, leucine, and iso-leucine, is also regulated by the gut microbiota and is necessary for a proper immune response (Man et al., 2020; Agus et al., 2021). BCAAs play a role in Treg cell proliferation via mammalian targets of rapamycin C1-dependent pathways and in antibody production (Ikeda et al., 2017; Man et al., 2020; Martín and Ramos, 2021). Lipoic acid is an antioxidant reported to reduce oxidative stress during infection via upregulation of Nf-e2-related factor 2 (Nrf2) (Qiao et al., 2013; Lu et al., 2019). Nrf2 has a protective function against SAR-CoV-2 infection (Bousquet et al., 2020; Omarjee et al., 2020). In addition, we also observed upregulation of KEGG pathways related to the production of SCFA in immunized mice, such as citrate cycle (TCA cycle), selenocompound metabolism, alanine, aspartate, and glutamate metabolism, geraniol degradation, lysine degradation, and butanoate metabolism (Tsukuda et al., 2021; Zhang et al., 2021; Frolova et al., 2022). These pathways also have strong correlations with immunization-enriched commensals, as well as immune response further indicating the role of SCFA and SCFA-producing bacteria in immune response to vaccination.

Our study has limitations. First, baseline microbiome composition was not identified in this study owing to limited population size. As described in previous studies, baseline microbiome greatly influences response to vaccines. Secondly, the association of the gut microbiome and its metabolites was only measured with antibody and cytokines response. The association of other effector molecules with the gut microbiome can further our insight on the immunogenicity of LAB-based vaccines. Finally, the gut microbiome of low and high responders to the LAB-based mucosal vaccine was not described and compared in this study. Although our findings offer valuable insights on the response of the gut microbiome to mucosal immunization with LAB-based vaccine, these limitations must be addressed in future studies to design a more effective LAB-based mucosal vaccine against SARS-CoV-2.

## Conclusion

Overall, we demonstrated in this study that mucosal immunization with recombinant *L. plantarum* SK156 expressing SARS-CoV-2 spike S1 epitopes altered the gut microbiome composition and function and SCFA levels in mice. Moreover, these changes are strongly associated with an enhanced immune response to mucosal vaccines. Enrichment of beneficial gut bacteria, such as Muribaculaceae, *Mucispirillum*, Ruminococcaceae, *Alistipes, Roseburia*, and Clostridia vadinBB60, may play a role in improved antibody and anti-inflammatory cytokine production through altered metabolism and butyrate production. Our findings provide further insights on the role of the gut microbiome in host’s immune response to vaccines, especially a novel mucosal vaccine against SARS-CoV-2. Finally, additional correlative studies between the baseline microbiome and other markers of immune response to COVID-19 vaccines are needed to improve immunogenicity and fully assess the efficacy of LAB-based vaccines.

## Data availability statement

The datasets presented in this study can be found in online repositories. The names of the repository/repositories and accession number(s) can be found in the article/**Supplementary Material**.

## Author contributions

Conceptualization: D-KK, LE and VDV. Methodology: I-CH, VDV, RV. Formal analysis and investigation: I-CH, RV and JHS. Writing – original draft: I-CH and RV. Visualization: RV. Writing- review and editing: D-KK, LE and VV. Funding acquisition: D-KK and LE. Supervision: D-KK, LE and VDV. All authors contributed to the article and approved the submitted version.
