# Supplementary material for: Alterations in the gut microbiome and its metabolites are associated with the immune response to mucosal immunization with Lactiplantibacillus plantarum-displaying recombinant SARS-CoV-2 spike epitopes in mice
Source: Front Cell Infect Microbiol. 2023 Aug 29;13:1242681. doi: 10.3389/fcimb.2023.1242681 (PMC10495993; doi:10.3389/fcimb.2023.1242681)
Supplement: Supplementary file 1 [file DataSheet_1.pdf]

Supplementary Material

Alterations in the gut microbiome and its metabolites are associated with the immune response to mucosal immunization with *Lactiplantibacillus plantarum*-displaying recombinant SARS-CoV-2 spike epitopes in mice

In-Chan Hwang[1†], Robie Vasquez[1†], Ji Hoon Song[1], Lars Engstrand[2], Valerie Diane Valeriano[2], and Dae-Kyung Kang[1]*

*** Correspondence:**
Dae-Kyung Kang
dkkang@dankook.ac.kr

[†]These authors contributed equally to this work and share first authorship

Table S1. Primers used for sequencing of V3-V4 hypervariable regions of 16S rRNA (MiSeq).

Primer	Sequence
341F	CCTACGGGNGGCWGCAG
805R	GACTACHVGGGTATCTAATCC

Table S2. Reads data before and after processing.

Index		Control	SK156	S1-1	S1-2	S1-3	S1-4	Total
Raw reads	mean	162765	182941.2	135593.3	145286	148482	137964.9	7253311
	SD	7705.44	18068.93	21878.97	19886.17	28133.49	20686.57	148026.8
Valid reads	mean	48920	69729.40	73186	79317.9	87926.66	76686.90	3676495
	SD	13759.18	14511.64	12356.48	10411.94	16943.29	10969.78	75030.51

Control, n=5; SK156, n=5; S1-1, n=10; S1-2, n=10; S1-3, n=9; S1-4, n=4.

Table S3. Alpha diversity measures.

**Index**		**Control**	**SK156**	**S1-1**	**S1-2**	**S1-3**	**S1-4**	***p* value**
Chao1 (Richness)	mean	106.8[a]	165.6[a]	537.9[b]	581.2[b]	571.7[b]	566.3[b]	0.0002
	SD	58.89	44.89	89.95	109.2	87.80	72.47	
Shannon (Diversity)	mean	4.390[a]	5.207[a]	6.678[b]	6.676[b]	6.906[b]	6.825[b]	0.0006
	SD	1.677	1.085	0.5748	0.4647	0.2404	0.3112	
Pielou's Evenness	mean	0.6549	0.7105	0.7444	0.7387	0.7652	0.7550	0.8409
	SD	0.1997	0.1289	0.05680	0.03413	0.03049	0.02754	

Control, n=5; SK156, n=5; S1-1, n=10; S1-2, n=10; S1-3, n=9; S1-4, n=4.
P value is calculated using Kruskal-Wallis test with *post hoc* Dunn's multiple comparisons test.
Different superscripts on mean values denote significant difference. *P* value is significant at < 0.05.
SD is standard deviation.

Table S4. PERMANOVA pairwise result for Bray-Curtis distance matrix.

Group 1	Group 2	Sample size	Permutations	pseudo-F	p-value	q-value
Control	S1-1	15	999	5.125554	0.001	0.001667
Control	S1-2	15	999	5.265969	0.002	0.0025
Control	S1-3	14	999	6.333999	0.001	0.001667
Control	S1-4	15	999	6.372894	0.001	0.001667
Control	SK156	10	999	1.553906	0.188	0.188
S1-1	S1-2	20	999	2.12212	0.001	0.001667
S1-1	S1-3	19	999	3.384528	0.001	0.001667
S1-1	S1-4	20	999	2.978561	0.001	0.001667
S1-1	SK156	15	999	2.915815	0.001	0.001667
S1-2	S1-3	19	999	4.1485	0.001	0.001667
S1-2	S1-4	20	999	1.771069	0.012	0.012857
S1-2	SK156	15	999	2.706474	0.004	0.004615
S1-3	S1-4	19	999	4.217082	0.001	0.001667
S1-3	SK156	14	999	3.459629	0.002	0.0025
S1-4	SK156	15	999	3.659585	0.002	0.0025

Table S5. PERMANOVA pairwise result for weighted uniFrac.

Group 1	Group 2	Sample size	Permutations	pseudo-F	p-value	q-value
Control	S1-1	15	999	6.2201510	0.003	0.0045
Control	S1-2	15	999	5.6719838	0.001	0.00214285
Control	S1-3	14	999	9.2634398	0.001	0.00214285
Control	S1-4	15	999	7.7232928	0.001	0.00214285
Control	SK156	10	999	1.0415956	0.367	0.39321428
S1-1	S1-2	20	999	2.5137987	0.009	0.01125
S1-1	S1-3	19	999	5.4520774	0.001	0.00214285
S1-1	S1-4	20	999	3.3190204	0.001	0.00214285
S1-1	SK156	15	999	3.7245649	0.004	0.00545454
S1-2	S1-3	19	999	6.1357333	0.001	0.0021428
S1-2	S1-4	20	999	1.0324704	0.4	0.4
S1-2	SK156	15	999	2.2824841	0.035	0.04038461
S1-3	S1-4	19	999	6.1306237	0.001	0.00214285
S1-3	SK156	14	999	4.4878222	0.003	0.0045
S1-4	SK156	15	999	3.6779437	0.003	0.0045

Table S6. Relative abundance (in %) at phylum and genus level. (cut-off set at >0.1%)

Taxa	Control	SK156	S1-1	S1-2	S1-3	S1-4	*p* value
**Phylum**							
Firmicutes	80.86	73.94	63.52	62.74	61.73	62.73	0.004
Bacteroidota	10.41	15.24	29.86	25.59	25.69	25.78	0.005
Desulfobacterota	2.38	4.55	2.11	5.16	5.14	4.23	0.009
Campylobacterota	4.12	4.67	3.10	4.08	4.16	2.93	0.62
Deferribacterota	1.89	1.40	0.54	1.43	2.38	3.01	0.001
Actinobacteriota	0	0	0.47	0.64	0.50	0.77	0.001
Patescibacteria	0.30	0.20	0.39	0.36	0.31	0.52	0.39
Other phyla	0.04	0.01	0.01	0.01	0.08	0.04	
**Genus**							
Lachnospiraceae NK4A136 group	42.52	28.61	20.72	20.52	15.35	19.81	0.01
Muribaculaceae	2.21	3.92	10.94	11.06	7.83	11.31	< 0.001
Lachnospiraceae unclassified	7.51	8.87	7.23	8.08	11.82	9.17	0.33
Lactobacillus	10.25	10.41	8.84	9.43	5.79	8.14	0.67
Alistipes	3.84	6.09	8.06	9.14	7.60	9.61	0.01
Lachnospiraceae uncultured	2.39	1.93	3.74	4.02	5.01	4.15	0.01
Desulfovibrio	2.36	4.44	1.47	4.96	4.67	3.92	0.004
Helicobacter	4.12	4.67	3.10	4.08	4.16	2.93	0.42
Odoribacter	0	0.03	3.71	0.13	7.18	0.86	< 0.001
Rikenellaceae RC9 gut group	1.15	1.53	3.14	2.46	1.77	2.34	0.003
Bacteroides	3.10	3.45	2.79	2.34	0.64	1.07	0.04
Limosilactobacillus	1.78	3.46	1.68	1.77	1.12	1.57	0.07
Mucispirillum	1.89	1.40	0.54	1.43	2.38	3.01	< 0.001
Anaerotruncus	0.65	0.71	1.99	1.75	1.53	1.39	0.09
Clostridia vadinBB60 group	0.25	0.93	1.20	1.14	2.98	1.34	0.001
Lachnoclostridium	1.46	1.24	1.39	1.35	1.64	1.25	0.79
[*Eubacterium*] *xylanophilum* group	2.09	1.54	1.79	1.27	0.99	1.00	0.29
Oscillospiraceae uncultured	0.89	0.95	1.30	1.23	2.20	1.37	0.03
Roseburia	0.16	0.83	2.09	1.28	1.57	1.05	0.009
Ligilactobacillus	2.86	1.59	1.76	0.91	0.30	0.86	0.01
Clostridia UCG-014	2.45	1.17	1.04	0.93	0.95	1.29	0.85
Lachnospiraceae UCG-006	1.28	1.88	0.57	0.72	1.22	1.24	0.15
Ruminococcaceae uncultured	0.19	0.45	0.73	0.75	1.43	0.99	< 0.001
Incertae Sedis	0.65	0.66	0.64	0.73	0.86	0.67	0.99
Oscillospiraceae unclassified	0.07	0.52	0.48	0.63	0.83	0.71	< 0.001
Other genera	3.88	8.74	9.06	7.88	8.19	8.96	

Control, n=5; SK156, n=5; S1-1, n=10; S1-2, n=10; S1-3, n=9; S1-4, n=10.

A.

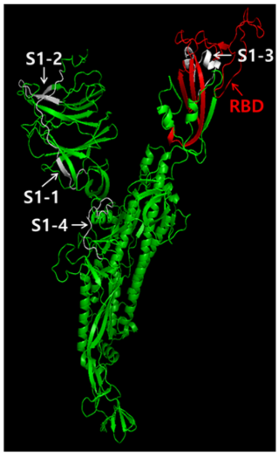

B.

Epitope	Protein sequence	DNA sequence (5'-3')
S1-1	QCVNLTTRTQLPPAYTNSFTRGV	CAATGTGTTAATTTAACGACGCGGACC<br>CAATTACCACCGGCTTATACGAATAGT<br>TTTACGCGGGGTGTT
S1-2	FLGVYYHKNNKSWMES	TTTTTAGGTGTTTACTATCATAAGAATA<br>ACAAGAGTTGGATGGAAAGT
S1-3	GDEVRQIAPGQTGKIADYNYKLP	GGTGATGAAGTTCGGCAAATTGCGCC<br>AGGTCAAACGGGTAAGATTGCTGATTA<br>TAATTATAAGTTACCAGAT
S1-4	ADQLTPTWRVYSTGSNVF	GCCGATCAATTAACGCCAACGTGGCG<br>GGTTTATAGTACCGGTAGTAATGTTTT<br>T

Figure S1. **The SARS-CoV-2 Spike 1 epitopes**. 3D structure of the epitopes which corresponds to the SARS-CoV-2 protein (A). Proteins and optimized DNA sequences of SARS-CoV-2 Spike 1 epitopes. From Hwang et al., 2023 (doi: 10.1186/s12934-023-02100-7).

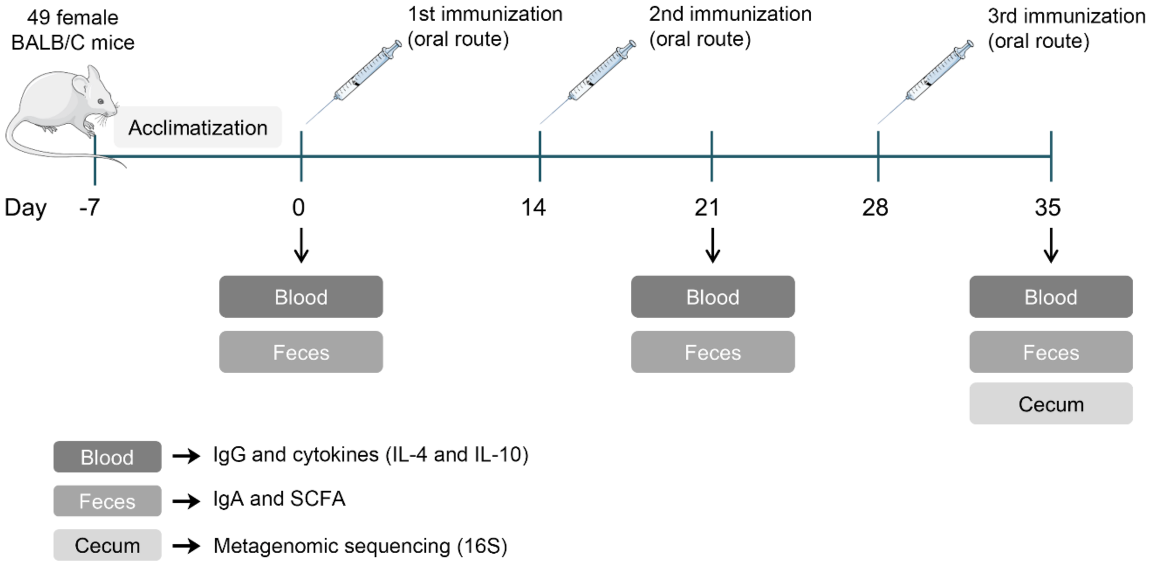

Figure S2. **Immunization strategy for the BALB/C mice.** Forty-nine BALB/c mice were randomly divided into 6 groups and immunized orally three times at days 0, 21, and 28 after 1 week of acclimatization. Blood and feces were collected on day 0 for pre-immunization data. At days 21 and 35 blood and feces samples were collected for antibody/cytokine analysis and SCFA quantification. Additionally, ceca were collected at day 35 for 16S amplicon metagenomic sequencing.

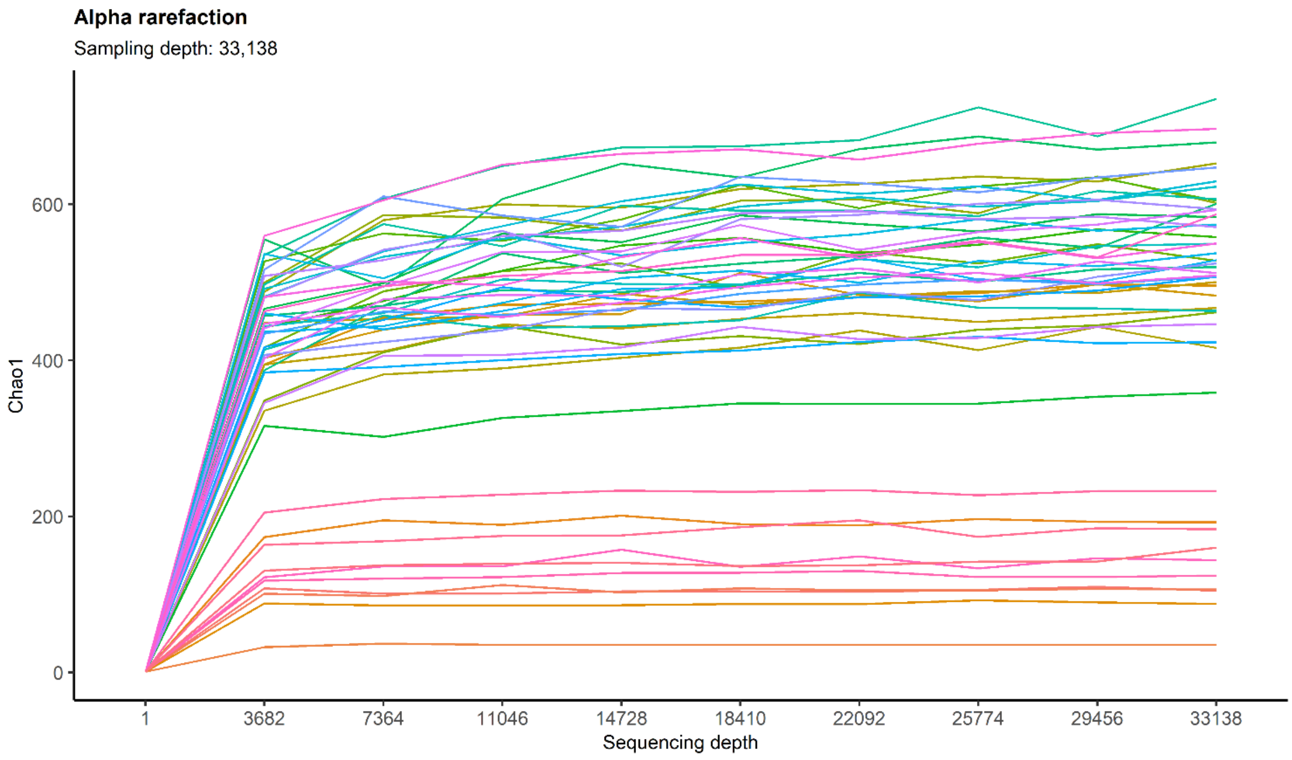

Figure S3. **Rarefaction curve.** Alpha-rarefaction curve showing Chao1 index at several sequencing depths, with plateau at 33,138 sequences.

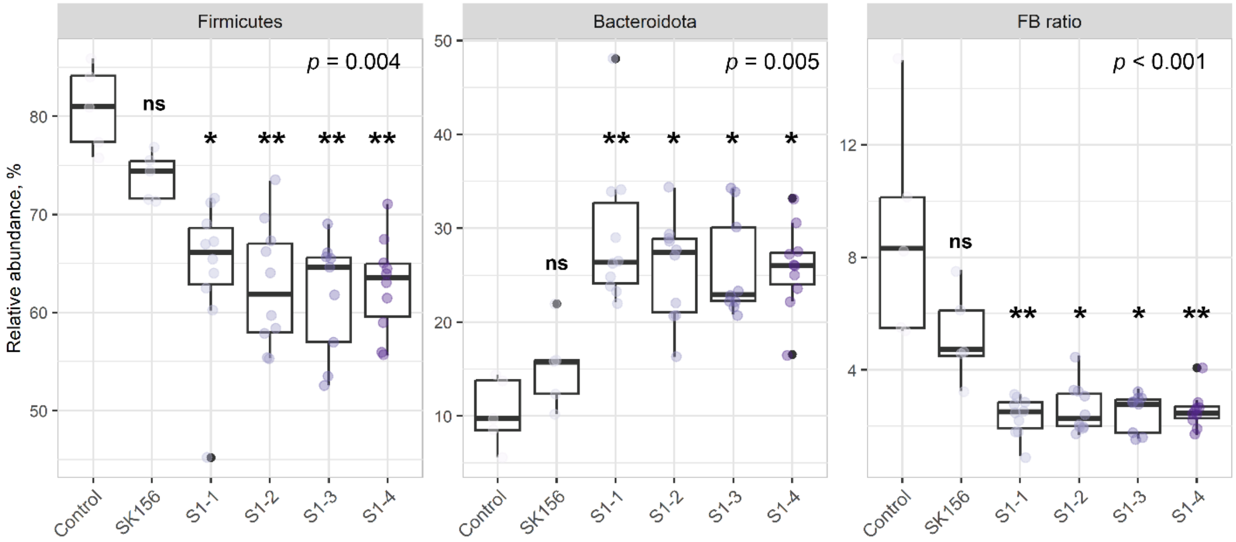

Figure S4. Relative abundances of phyla Firmicutes and Bacteroidota, and their ratio (Firmicutes:Bacteroidota).

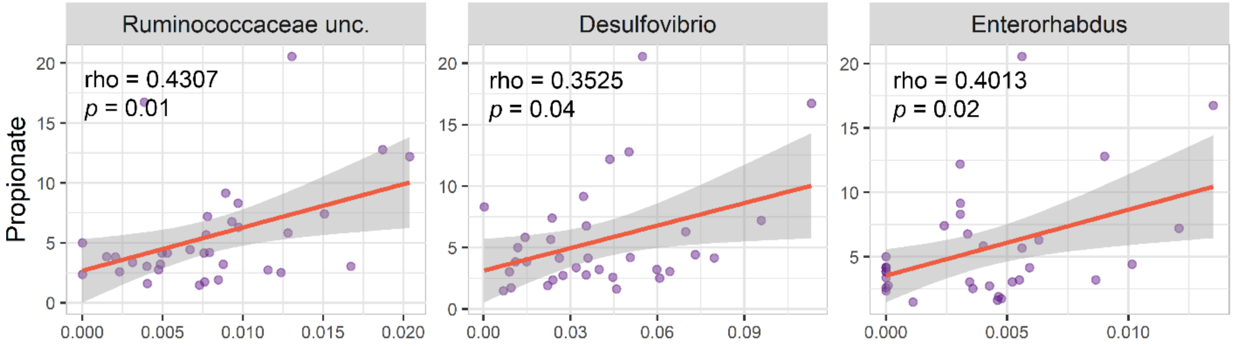

Figure S5. Correlation between fecal propionate and abundance of taxonomic markers.

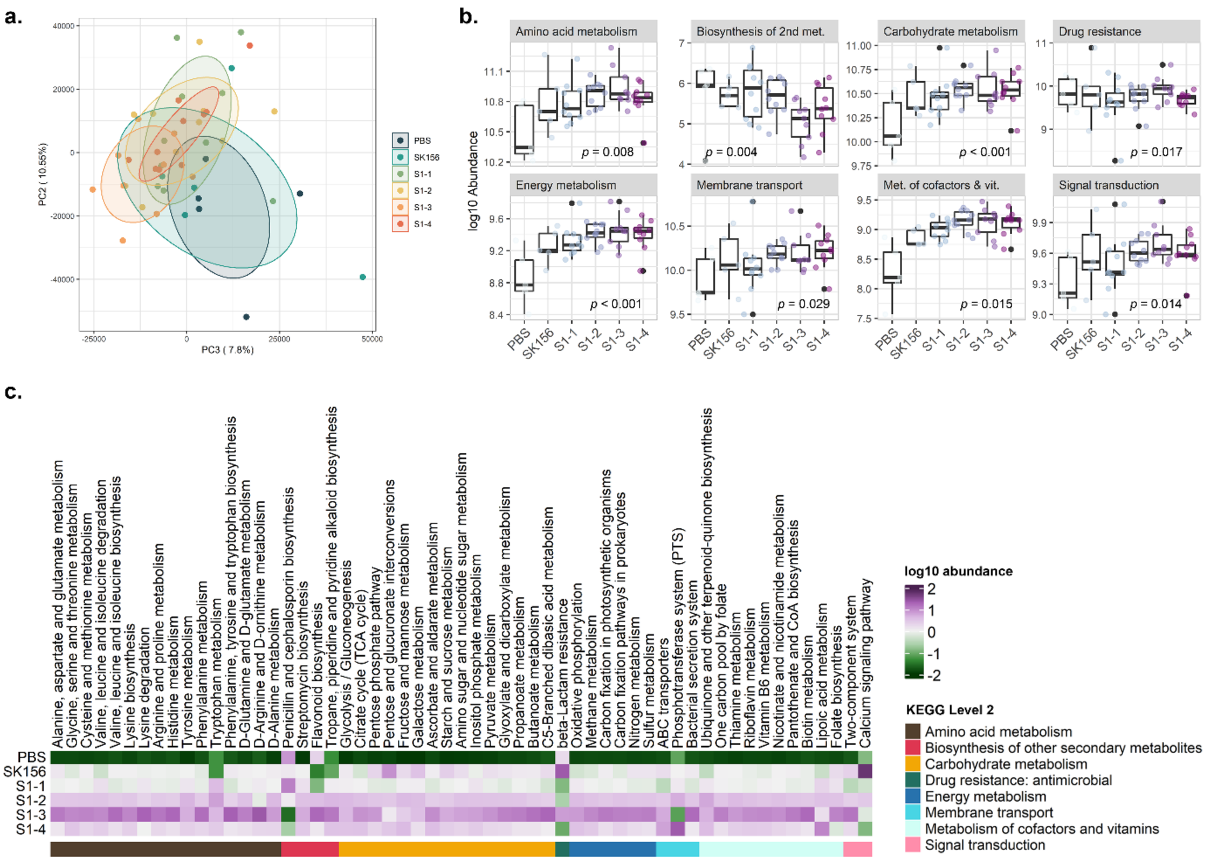

Figure S6. Predicted KEGG pathway enrichment using PICRUSt2. Principal component analysis (PCA) (A). Abundance (log10) of selected KEGG pathways at level 2 (B). KEGG pathways related to metabolism (level 3) (C).
